# Patterns of hormonal contraception use in women with sickle cell disease and employer-sponsored insurance^☆^

**DOI:** 10.1016/j.contraception.2026.111399

**Published:** 2026-01-28

**Authors:** Sarah H. O’Brien, Sherraine L. Della-Moretta, Joseph R. Stanek, Susan E. Creary, Andrea H. Roe, Wendy Xu, Natasha Bala, Sara K. Vesely, Rose Y. Hardy

**Affiliations:** a Division of Pediatric Hematology/Oncology/BMT, Nationwide Children's Hospital and The Ohio State University College of Medicine, Columbus, OH, United States; b Center for Child Health Equity and Outcomes Research, Abigail Wexner Research Institute at Nationwide Children's Hospital, Columbus, OH, United States; c Division of Hematology, The Ohio State University College of Medicine, Columbus, OH, United States; d Biostatistics Resource at Nationwide Children's Hospital, Columbus, OH, United States; e Department of Obstetrics and Gynecology, University of Pennsylvania, Perelman School of Medicine, Philadelphia, PA, United States; f Division of Health Services Management and Policy, The Ohio State University, College of Public Health, Columbus, OH, United States; g Division of Pediatric Hematology/Oncology/BMT, Phoenix Children’s Hospital, The University of Arizona College of Medicine, Phoenix, AZ, United States; h Department of Biostatistics and Epidemiology, Hudson College of Public Health, The University of Oklahoma, Oklahoma City, OK, United States

**Keywords:** Contraception, Epidemiology, Intrauterine device, Sickle cell anemia, Women

## Abstract

**Objective:**

While an improved understanding of the epidemiology and outcomes of hormonal contraceptive use in persons with sickle cell disease is needed to better inform evidence-based guidelines, patterns of contraception use remain understudied. Our objective was to examine patterns of contraception use among patients with sickle cell disease and employer-sponsored insurance.

**Study design:**

Our analyses utilized the Merative^™^ MarketScan^®^ Commercial Database (2012–2019). The study included females 12–44 years meeting the study definition of sickle cell disease (presence of three ICD-9 or ICD-10 codes consistent with sickle cell disease). We utilized pharmacy and procedure claims to capture hormonal contraception use.

**Results:**

We identified 5957 unique females of reproductive age (12–44 years) with sickle cell disease, 1653 (28%) of whom utilized hormonal contraception. When examining the first identified contraception prescription, 43.9% were prescribed estrogen-containing agents and 56.1% were prescribed progestin-only. The most frequently prescribed contraceptive was combined contraceptive pills (n = 645, 39%), followed by depot medroxyprogesterone acetate (n = 413, 25%). Intrauterine devices and subcutaneous implants were prescribed to 15.7% and 6.4% of individuals, respectively.

**Conclusions:**

Our work identified hormonal contraception use in less than one-third of reproductive-age females with sickle cell disease using employer-sponsored insurance. Over 40% of new users of hormonal contraception received prescriptions for estrogen-containing agents, demonstrating a need for education regarding the 2024 U.S. MEC recommendations, which contradict estrogen for patients with sickle cell disease. Future directions should also include understanding patient characteristics and health outcomes among combined hormonal contraceptive users with sickle cell disease to better inform eligibility criteria.

**Implications:**

Estrogen-containing agents make up a substantial percentage of contraception prescriptions in women with sickle cell disease and employer-sponsored insurance. High-quality data regarding the safety of hormonal contraception in the setting of sickle cell disease are needed to inform evidence-based guidelines and medical eligibility criteria.

## 1. Introduction

The availability of safe and effective contraception is a critical component of comprehensive care for reproductive-aged women living with sickle cell disease [1]. While improved understanding of the epidemiology and outcomes of hormonal contraceptive use in persons with sickle cell disease is needed to better inform evidence-based guidelines, patterns of hormonal contraception use remain under studied, with most studies limited to single sites with small sample sizes. Investigators at the University of Pennsylvania sought to compare contraceptive use in patients with sickle cell disease at their institution to data from the National Survey of Family Growth [2]. Results demonstrated that hormonal contraception use in sickle cell disease was not only lower than that of the general population, but also differed in the types of contraceptive methods most commonly used. Survey data from the Johns Hopkins University Sickle Cell Center showed a lack of patient knowledge surrounding the efficacy of contraceptive methods, especially long-acting reversible contraceptives which respondents believed were less effective than data have shown [3]. Over half (58%) of the respondent population had experienced at least one unintended pregnancy. These studies suggest that hormonal contraception use in patients with sickle cell disease differs from the general population, although there are not nationwide data to corroborate this hypothesis.

One safety concern for use of contraception in women with sickle cell disease is the clearly established risk of thrombosis with estrogen-containing contraceptives in the general population [4]. However, while persons with sickle cell disease are at higher risk of thrombosis than the general population, and the use of combined hormonal contraception may further elevate this risk, there is very low certainty in the evidence on risk of contraception-related thrombosis for persons with sickle cell disease [5].

Our group has previously used Centers for Medicare and Medicaid Services data to assess patterns of hormonal contraception use in patients with sickle cell disease [6]. In a cohort of 7173 women with a new prescription or procedure for hormonal contraception, we found higher than expected prescribing of estrogen-containing contraception (44.6% of new contraception users), most commonly in the form of combined oral contraceptive pills. However, this study was limited by statewide variability in medication reporting, leading to an inability to examine days supplied in these prescriptions. Among users of progestin-only contraception, depot medroxyprogesterone acetate was the most used agent, while approximately 10% of new contraception users initiated an intrauterine device or implant.

The MarketScan databases, compiled and maintained by Truven Health Analytics, provide claims information for individuals with employer-sponsored insurance [7]. This resource addresses the limitations of Medicaid data by providing complete longitudinal pharmaceutical data and, therefore, a more accurate description of hormonal contraception patterns. We hypothesized that we would again find a high volume of estrogen-containing prescriptions among women with sickle cell disease utilizing hormonal contraception.

## 2. Materials and methods

### 2.1. Data source

Our analyses utilized the Merative^™^ MarketScan^®^ Commercial Database (2012–2019) [8]. This resource includes de-identified administrative claims data for individuals covered by any of over 300 employers that sponsor private health insurance, as well as data on their spouses and dependents. Each patient was assigned an encrypted, unique identification number which allows for linkage of all inpatient, outpatient, and pharmacy records over the duration of the study period. The use of de-identified administrative claims data is deemed exempt by the Institutional Review Board at Nationwide Children’s Hospital. Data were analyzed by R.H. and only R.H. had access to the primary data source. Information about diagnoses and/ or procedures was obtained using International Classification of Diseases-9 and 10, Current Procedural Terminology and Healthcare Common Procedure Coding System codes (Supplementary Table 1).

### 2.2. Study population and hormonal contraception

The study included females 12–44 years with sickle cell disease. Sex is determined at entry into the MarketScan database by patient-level demographic information and defined as male or female [9]. Sickle cell disease was defined as the presence of ≥ 3 international classification of disease-9 or international classification of disease-10 codes consistent with sickle cell disease, an algorithm that has demonstrated high sensitivity (96%), high positive predictive value (89%), and reasonable negative predictive value (68%) for identifying individuals with sickle cell disease [10,11]. Sickle cell disease genotype was identified with international classification of disease codes – an individual’s genotype was assigned as the most used genotype for each patient. If a patient had two different genotypes documented equally often, they were categorized as non-specific sickle cell disease. Severe sickle cell disease was defined as ≥ 3 inpatient admissions that included a code for sickle cell disease during the first full calendar year the individual was in the MarketScan database [12,13].

In reporting contraception use and method by calendar year, an individual was identified as a user of hormonal contraception if they had at least one pharmacy claim for oral, transdermal, vaginal, or injectable contraceptives for short-acting contraceptives. Individuals with pharmacy claims for more than one type of short-acting contraceptives in a given calendar year were coded as using the method that was in use for the longest period in that year. Long-acting reversible contraceptive use was analyzed using pharmacy and procedure codes. If no removal codes were detected following intrauterine device or implant placement, and the individual remained enrolled in the insurance plan, we assumed the long-acting reversible contraceptive remained in place.

Combined hormonal contraception included combination oral contraceptive pills, transdermal patches, and the vaginal ring. Progestin-only contraception included the progestin-only pill or injectable depot medroxyprogesterone acetate. The long-acting reversible contraceptive category included subdermal implant or intrauterine device (both hormonal and copper versions). Oral medroxyprogesterone was not included in the analysis because this medication is indicated for menstrual disorders rather than as an effective method of contraception.

In conjunction with insurance eligibility data, prescription fill dates, and the days supplied field, we identified prescription episodes, length of time on short-acting hormonal contraception, and discontinuation. We defined discontinuation as 120 days (4 months) elapsing without a refill for short-acting contraceptives. We defined discontinuation of depot medroxyprogesterone acetate as 196 days (16 weeks) without a repeat injection [14]. To estimate the duration of use for long-acting reversible contraceptives, we utilized procedure codes for removal of these devices, or procedure codes for insertion of another intrauterine device or implant. We performed a sub-analysis of long-acting reversible contraceptive users with 24 months of continuous enrollment after long-acting reversible contraceptive placement to calculate the percentage with removal codes at 6-, 12-, and 24-months. We defined contraceptive switching as changes in contraception method type (e.g., combined hormonal contraception to intrauterine device, progestin-only pills to combined hormonal contraception).

### 2.3. Statistical analysis

Descriptive statistics were utilized to report the percentage of contraception users in each calendar year of the database. To aid in protecting the privacy of beneficiaries, if the number of observations was ≤ 11 in any category, we did not report exact frequencies. Duration of contraception use in days was summarized as median and interquartile range. Univariable comparisons of demographic and clinical characteristics between combined hormonal contraception, progestin-only contraception, and long-acting reversible contraceptive users were completed using appropriate nonparametric methods, such as chi-square, Wilcoxon rank-sum, or Kruskal-Wallis tests. Data cleaning and statistical analyses were conducted in SAS 9.4 and Stata 14.0. Figures were prepared in PowerPoint. P-values < 0.05 were considered statistically significant.

## 3. Results

Between 2012–2019, we identified 5957 unique women of reproductive age in the MarketScan database meeting the study definition of sickle cell disease, of whom 59.9% were identified as hemoglobin (Hb)SS. Within the population of women with sickle cell disease, there was a similar distribution among age groups (12–18, 19–26, 27–34, and 35–44 years, Supplementary Table 2). More than half of the individuals were from the South geographic region, with the fewest living in the West. Fewer than 10% of women were classified as residing in a rural area, defined as a county not classified as a Metropolitan Statistical Area.

We identified 1653 (27.7%) unique users of hormonal contraception (median continuous enrollment = 36 months, interquartile range: 22–65). The most frequently prescribed contraceptive was combination contraceptive pills (*n* = 645, 39%), followed by depot medroxyprogesterone acetate (*n* = 413, 25%) (Table 1). Long-acting reversible contraceptives such as intrauterine device and implants were the first-identified contraception claim in 15.7% and 6.4% of individuals, respectively. There were no significant differences in contraceptive method prescribing practices between metro and non-metro residence. There were differences noted in median age at time of first identified contraceptive prescription or procedure, with a lower percentage of adolescents utilizing long-acting reversible contraceptives compared to combined hormonal contraception and other progestin-only contraception. A difference in prescribing practices based on sickle cell disease genotype was also identified. For example, 62% of progestin-only contraception users had HbSS as compared to 49% of combined hormonal contraception users (Table 1). Among progestin-only contraception users and long-acting reversible contraceptive users, 10% met the study definition of severe sickle cell disease, as compared to 6% of the combined hormonal contraception users.

When examining the total population of female beneficiaries with sickle cell disease in each calendar year, contraceptive use and method mix remained stable over the study period, with 16–25% of women using any type of hormonal contraception each year (Table 2). Combination oral contraceptive pills were the most prescribed short-acting method of contraception, with 8–10% of women using this method each year, followed by the depot medroxyprogesterone acetate injection. Use of long-acting reversible contraceptives (implant or intrauterine device), occurred in approximately 2–13% of women each calendar year, with use increasing over time. Although it does not contain a hormone, women with claims for a copper intrauterine device were also included in our description of contraception use, accounting for 9% (33 of 363) of identified intrauterine devices.

We examined median days’ supply as a proxy for the length of time individuals remained on a newly prescribed short-acting contraceptive (Table 3). Those receiving depot medroxyprogesterone acetate injections stayed on their prescribed contraceptive method the longest (median 180 days, interquartile range 90–390). Over 20% of long-acting reversible contraceptives (*n* = 499) were removed within 6 months of placement, while 41% were removed within two years of placement (Fig. 1).

Approximately half of the enrollees who used contraception (*n* = 894, 54%) changed their contraceptive type during the years of observation. Among women with identified switching, the most common type of switch was to discontinue an estrogen-containing contraceptive and switch to depot medroxyprogesterone acetate injections or an intrauterine device (Fig. 2). The second most common switch pattern was to change from depot medroxyprogesterone acetate injections to an estrogen-containing contraceptive.

## 4. Discussion

In a large cohort of women with sickle cell disease with employer-sponsored insurance, 16–25% of women in each calendar year had evidence of hormonal contraception use, similar to the prevalence of contraception use we identified in our analysis of women with sickle cell disease and Medicaid insurance [6]. Our results suggest that contraceptive use is comparable between women with sickle cell disease and national data for the general population of Black women. In the National Survey of Family Growth (2017–2019), 12.7% of reproductive-age Black or African American women (15–49 years of age) were currently using an oral contraceptive pill (as compared to 10–12% in the MarketScan population in 2017–19), and 14% were using a long-acting reversible contraceptive (as compared to 13% in MarketScan). Current use of depot medroxyprogesterone acetate was reported in 8.4% of Black or African American women (compared to 6% in MarketScan) [15].

Concerns about risk of thromboembolism among individuals with sickle cell disease recently led to changes in the Centers for Disease Control recommendations regarding hormonal contraception in this population. The 2024 Medical Eligibility Criteria, which previously categorized estrogen-containing contraception as Category 2 (benefits of using the method outweigh risks), now contraindicates these methods for those with sickle cell disease (Category 4) [16,17]. Progestin-only methods remain recommended for unrestricted use (Category 1), except for depot medroxyprogesterone acetate, which is classified as Category 2/3 due to its unknown risk-to-benefit ratio. While our analysis includes data collected well before (2012–2109) the 2024 Medical Eligibility Criteria updates, it is notable that combined oral hormonal agents were the most commonly prescribed contraception. In a survey of the hemoglobinopathy special interest group of the American Society of Pediatric Hematology and Oncology performed in 2019, 23 of 77 providers (30%) agreed or strongly agreed that combined hormonal contraception should never be used in adolescents or young adults with sickle cell disease, while 50% agreed or strongly agreed that combined hormonal contraception could be used if they do not carry other risk factors for stroke such as smoking, hypertension, or obesity [18].

Data on the thrombotic risk of estrogen-containing contraception in the sickle cell disease population remain limited. A systematic review of articles (through 2022) on sickle cell disease and adverse health outcomes associated with hormonal contraception identified only nine articles, with only three examining thrombosis [19]. The certainty for all outcomes was determined to be very low with serious concerns for bias. More recently, an analysis of individuals with sickle cell disease insured by Medicaid did not reveal differences in thrombosis risk between those prescribed estrogen-containing as compared to progestin-only products, but this work was limited by missing data regarding duration of use for short-acting contraceptives [6].

Our analysis revealed a discontinuation rate of approximately 30% at one year, and 40% at two years after insertion of long-acting reversible contraceptive methods. In a large retrospective study of women undergoing intrauterine device placement at an urban academic medical center (*n* = 956), 35% of women had discontinued levonorgestrel intrauterine device at two years compared to 42% of copper intrauterine device users [20]. Our analysis is limited as it was restricted to participants with a full 2-years of enrollment after long-acting reversible contraceptive placement, and our data source did not allow us to evaluate the reasons behind early discontinuation of long-acting reversible contraceptives. Among short-acting contraceptives, depot medroxyprogesterone acetate injections had the longest duration of use with a median of 6 months of use (interquartile range 3–13 months), as compared to oral progestin-only pills, which had a median use of less than 4 months. The median MarketScan enrollment period for contraception users was 36 months, and approximately half of contraception users switched contraceptive type during that timeframe. The most common switch was from an estrogen-containing contraceptive to a progestin-only form (typically depot medroxyprogesterone acetate or intrauterine device).

Limitations of our work include a study population of women with sickle cell disease and employer-sponsored insurance, which reflects a minority of the national US population of individuals with sickle cell disease. In a claims-based analysis of patients with sickle cell disease receiving frequent red blood cell transfusions (≥6 transfusions in any 12-month period), those covered by commercial insurance had a lower prevalence of clinical complications and less healthcare resource utilization as compared to those covered by Medicaid [21], which may impact contraception prescribing patterns (for example, health care providers may have a greater level of comfort prescribing an estrogen-containing product to a patient with sickle cell disease without complications). The use of administrative claims data relies on coding and documentation by providers, which is not always accurate. In particular, the use of international classification of disease codes for the identification of sickle cell disease genotype has low accuracy [22,23] and our methodology might have led some patients with less severe genotypes to be misclassified as having HbSS.

We chose to focus our analyses on first-identified contraceptive prescription rather than contraceptive with longest history of use in the database. This choice may bias our analysis towards an increased number of prescriptions for short-acting contraceptives. We are also unable to ascertain the shared decision-making process for the use of estrogen-containing versus progestin-only contraception in any beneficiary, nor were we able to determine all potentially informative medical history.

In conclusion, our work demonstrates that contraceptive uptake in reproductive-age women with sickle cell disease and employer-sponsored insurance is comparable to the national United States population of Black women. There is an urgent need to better understand contraceptive safety and risks in women with sickle cell disease [24,25]. While there are increasing concerns about the safety of estrogen-containing contraception in women with sickle cell disease, this type of contraception was used by approximately 40% of new users of hormonal contraception prior to the new recommendations contraindicating estrogen, demonstrating a need for education regarding the new U.S. MEC recommendations. However, future directions should also include understanding patient characteristics and health outcomes among hormonal contraceptive users with sickle cell disease to inform eligibility criteria better.

## Supplementary Material

1

## Figures and Tables

**Fig. 1.**
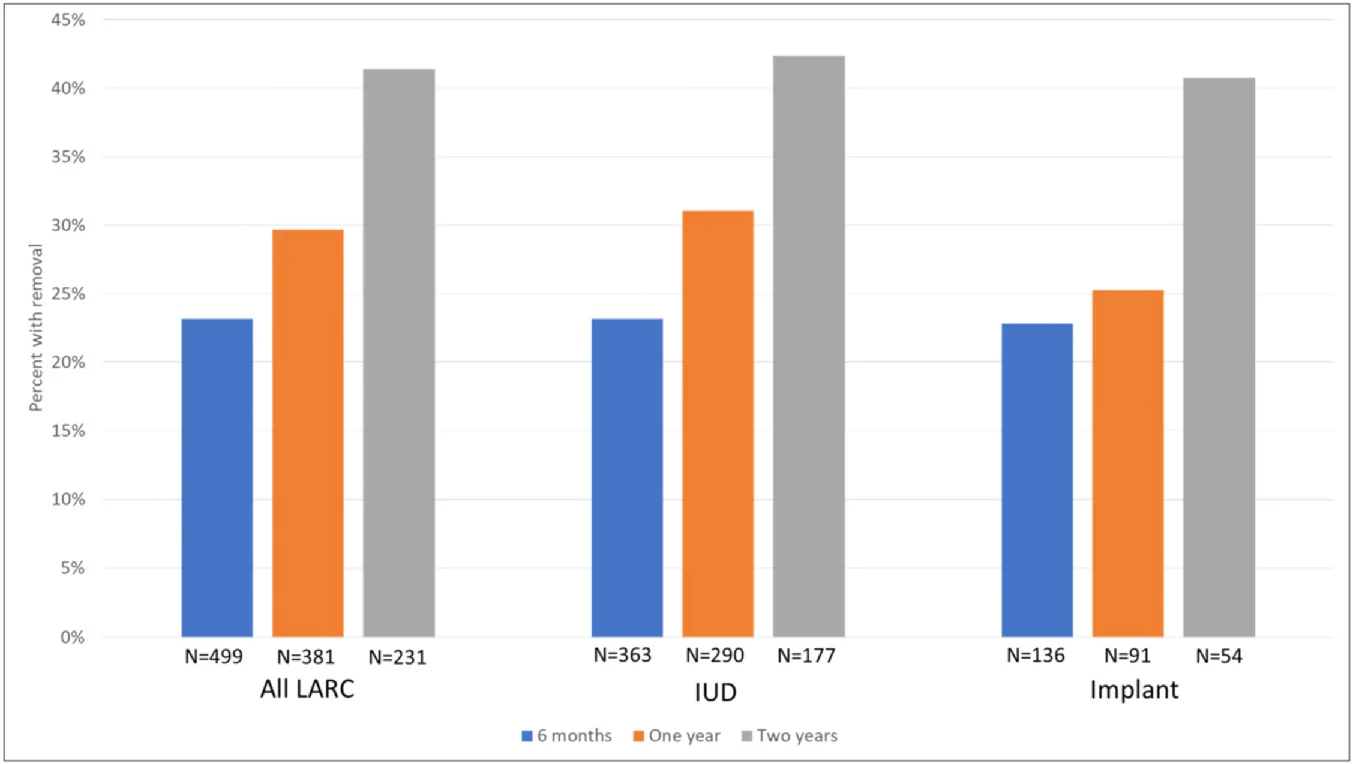
Long-acting reversible contraception (LARC) removal overall and by LARC method by 6 month-, 1-year, and 2-year observation period among reproductive-age women with SCD, MarketScan Database 2012–2019. Note: Observation period begins at LARC insertion date. Sample sizes indicate the total number of LARC episodes among women with enrollment windows at least as long as observation period. Abbreviations: IUD, intrauterine device; LARC, long-acting reversible contraception; SCD, sickle cell disease.

**Fig. 2.**
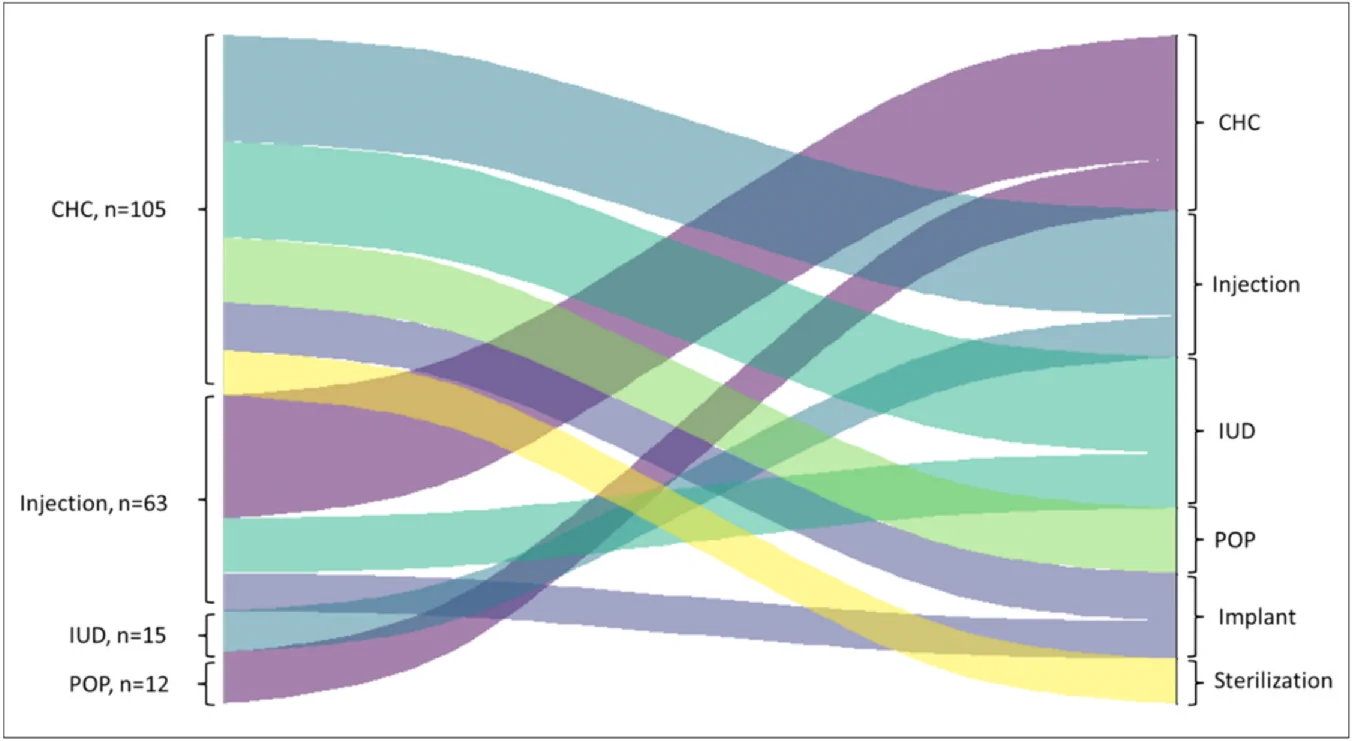
Contraceptive switching patterns among reproductive age women with SCD with at least one observed contraceptive switch occurring between 2012 and 2019, MarketScan database. Note: Patterns with less than 11 observations were omitted from this figure. Abbreviations: CHC, combined hormonal contraception; IUD, intrauterine device; POP, progestin-only pill; SCD, sickle cell disease.

**Table 1:** Demographic and Clinical Characteristics of CHC, POC, and LARC Users among First-Identified Contraception Claim in Reproductive-age Women with SCD (*n* = 5957), MarketScan Database 2012–2019

	Type of Contraceptive				
	
Characteristic	Total	CHC	POC (Pills and Depo Shots)	LARC Implant/IUD	p-Value

Unique Patients, *n* (% of all unique pts)	1653	726 (44)	563 (34)	364 (22)	
Mo, continuous enrollment, median (IQR)	36 (20; 63)	36 (22; 64)	36 (18; 60)	36 (24; 68)	0.11
SCD claims, median (IQR)	14 (5; 33)	10 (5; 26)	17 (6; 48)	16 (6; 40)	< 0.01
Age at First Contraceptive Claim (yrs), *n* (%)					
12–18	232 (14.0)	103 (14.2)	100 (17.8)	29 (8.0)	< 0.01
19–26	587 (35.5)	242 (33.3)	221 (39.3)	124 (34.1)	
27–34	497 (30.1)	223 (30.7)	146 (25.9)	128 (35.2)	
35–44	337 (20.4)	158 (21.8)	96 (17.1)	83 (22.8)	
Residence, *n* (%)					
Metro	1464 (93.2)	654 (93.7)	482 (91.8)	328 (94.5)	0.25
Non-Metro	106 (6.8)	44 (6.3)	43 (8.2)	19 (5.5)	
Geographic Region					
Northeast	256 (15.7)	103 (14.4)	79 (14.3)	74 (20.4)	0.01
North Central	232 (14.2)	91 (12.7)	85 (15.3)	56 (15.5)	
South	1047 (64.2)	469 (65.5)	366 (66.1)	212 (58.6)	
West	97 (5.9)	53 (7.4)	24 (4.3)	20 (5.5)	
Sickle cell genotype, *n* (%)					
Hb SS	890 (53.8)	354 (48.8)	347 (61.6)	189 (51.9)	< 0.01
Hb SC	294 (17.8)	132 (18.2)	84 (14.9)	78 (21.4)	
Hb Sβ^0/+^	168 (10.2)	84 (11.6)	42 (7.5)	42 (11.5)	
Nonspecific SCD	301 (18.2)	156 (21.5)	90 (16.0)	55 (15.1)	
Cumulative patient years	1635	717	555	363	
Yearly SCD severity, *n* (%)					
High	139 (8.5)	46 (6.4)	56 (10.1)	37 (10.2)	0.03
Low	1496 (91.5)	671 (93.6)	499 (89.9)	326 (89.8)	

P-values result from Anova, Kruskal-Wallis H Test, or Chi-square tests as appropriate.

Abbreviations: CHC, combined hormonal contraception; IQR, interquartile range; IUD, intrauterine device; LARC, long-acting reversible contraception; POC, progestin-only contraception; SCD, sickle cell disease

Yearly SCD Severity: High: ≥ 3 inpatient admissions that included a code for SCD during one calendar year

Nonspecific SCD: Patients with 3 or more codes for sickle cell disease, but contain codes for more than one genotype (for example HbSS and HbSC) so genotype cannot be determined

**Table 2:** Contraceptive Use by Year, 2012–2019 (percentage of women with SCD, ages 12–44, with prescription or procedure codes consistent with current use of hormonal contraception), MarketScan Database 2012–2019

Year	Base N	Any Hormonal Contraception	CHC	Patch	Ring	DMPA (injection)	POP	Implant	IUD

2012	2261	16%	8%	< 1%	1%	2%	2%	< 1%	2%
2013	2314	20%	8%	< 1%	1%	6%	2%	1%	3%
2014	2449	22%	9%	< 1%	1%	6%	2%	1%	5%
2015	1810	24%	9%	< 1%	1%	7%	2%	2%	7%
2016	1812	25%	10%	< 1%	1%	6%	2%	2%	8%
2017	1802	25%	10%	< 1%	1%	6%	2%	4%	9%
2018	1825	23%	8%	< 1%	1%	6%	2%	4%	9%
2019	1688	21%	8%	1%	< 1%	6%	3%	4%	9%

*n* = 5957 unique women, many women appear for multiple years.

Abbreviations: CHC, combined hormonal contraception (oral); DMPA, depot medroxyprogesterone acetate; IUD, intrauterine device; POP, progestin-only pills; SCD, sickle cell disease

**Table 3:** Median Days Covered during Contraceptive Episodes and Percent Discontinued among Reproductive-Age Women with SCD, MarketScan Database 2012–2019

	Type of Contraception				
	
Characteristic	CHC	Injection	POP	Patch	Ring

Prescription Episodes	953	581	244	45	115
Median Days’ Supply (IQR)	168 (56; 364)	180 (90; 390)	84 (56; 252)	136 (56; 296)	112 (56; 292)
Percent Discontinued	36.9%	32.2%	36.5%	22.2%	35.7%

Abbreviations: CHC, combined hormonal contraception; IQR, interquartile range; POP, progestin-only pill; SCD (sickle cell disease).

## Appendix A. Supplementary material

Supplementary data associated with this article can be found in the online version at doi:10.1016/j.contraception.2026.111399.

## References

[1] Leroy-Melamed M, McGann P, Van Doren L. How we approach contraception for adolescents and young adults with sickle cell disease. Pediatr Blood Cancer 2025;72(6):e31683. 10.1002/pbc.31683 40134115

[2] Roe AH, Lang B, McAllister A, Gaitors MC, Smith-Whitley K, Schreiber CA, Contraceptive use and preferences among females with sickle cell disease. Contraception 2022;105:42–5. 10.1016/j.contraception.2021.08.009 34418379

[3] Pecker LH, Hussain S, Lanzkron S, Tao X, Thaler K, Burke AE, Women with sickle cell disease report low knowledge and use of long acting reversible contraception. J Natl Med Assoc 2021;113(5):552–9. 10.1016/j.jnma.2021.05.005 PMC10206766 34119338

[4] Dragoman MV, Tepper NK, Fu R, Curtis KM, Chou R, Gaffield ME. A systematic review and meta-analysis of venous thrombosis risk among users of combined oral contraception. Int J Gynaecol Obstet 2018;141(3):287–94. 10.1002/ijgo.12455 PMC5969307 29388678

[5] Pecker LH, Burke A, Streiff MB, Lanzkron S, Naik RP. Progestin-only contraception is first line for sickle cell disease: a comment on “Hormonal therapies in females with blood disorders: thrombophilia, thrombosis, hemoglobinopathies, and anemias. Res Pract Thromb Haemos 2023;7(6). 10.1016/j.rpth.2023.102183 PMC10498431 37711908

[6] Bala NS, Stanek JR, Vesely SK, Cronin RM, Creary SE, Roe AH, Comparison of thromboembolism outcomes in patients with sickle cell disease prescribed hormonal contraception. Blood Adv 2023;7(20):6140–50. 10.1182/bloodadvances.2024013554 PMC10582388 37585480

[7] Kulaylat AS, Schaefer EW, Messaris E, Hollenbeak CS. Truven health analytics MarketScan databases for clinical research in colon and rectal surgery. Clin Colon Rect Surg 2019;32(01):054–60. 10.1055/s0038-1673354 PMC6327721 30647546

[8] Merative. [cited 2024 6/5/2024]; Available from: 〈https://www.merative.com/real-world-evidence〉.

[9] Subramanya V, Claxton JNS, Lutsey PL, MacLehose RF, Chen LY, Chamberlain AM, Sex differences in treatment strategy and adverse outcomes among patients 75 and older with atrial fibrillation in the MarketScan database. BMC Cardiovasc Disord 2021;21:1–12. 10.1186/s12872-021-02419-2 PMC8679994 34915858

[10] Snyder AB, Zhou M, Theodore R, Quarmyne M-O, Eckman J, Lane PA. Improving an administrative case definition for longitudinal surveillance of sickle cell disease. Public Health Rep 2019;134(3):274–81. 10.1177/0033354919839072 PMC6505322 30970223

[11] Singh A, Sontag MK, Zhou M, Dasgupta M, Crume T, McLemore M, Evaluating the discriminatory ability of the sickle cell data collection program’s administrative claims case definition in identifying adults with sickle cell disease: validation study. JMIR Public Health Surveill 2023;9:e42816. 10.2196/42816 PMC10365593 37379070

[12] Kinney TR, Helms RW, O’Branski EE, Ohene-Frempong K, Wang W, Daeschner C, Safety of hydroxyurea in children with sickle cell anemia: results of the HUG-KIDS study, a phase I/II trial. Blood 1999;94(5):1550–4. 10477679

[13] Charache S, Terrin ML, Moore RD, Dover GJ, Barton FB, Eckert SV, Effect of hydroxyurea on the frequency of painful crises in sickle cell anemia. N Engl J Med 1995;332(20):1317–22. 10.1056/NEJM199505183322001 7715639

[14] O’Brien SH, Koch T, Vesely SK, Schwarz EB. Hormonal contraception and risk of thromboembolism in women with diabetes. 10.2337/dc16-1534 Diabetes Care 2017;40(2):233–8. 10.2337/dc16-1534 27899496

[15] Centers for Disease Control and Prevention. Key Statistics from the National Survey of Family Growth - C Listing. 2022 June 29, 2022; Available from: https://www.cdc.gov/nchs/nsfg/key_statistics/c-keystat.htm.

[16] Curtis KM. US selected practice recommendations for contraceptive use, 2016. MMWR. Recommend Rep 2016;65. 10.15585/mmwr.rr6504a1 27467319

[17] Nguyen AT. US medical eligibility criteria for contraceptive use, 2024. MMWR. Recommend Rep 2024;73. 10.15585/mmwr.rr7304a1 PMC11315372 39106314

[18] Leroy-Melamed M, Jacob S, Shew ML, Kazmerski TM. Provider attitudes, preferences, and practices regarding sexual and reproductive health for adolescents and young adults with sickle cell disease. J Adolesc Health 2021;69(6):970–5. 10.1016/j.jadohealth.2021.06.008 PMC8612941 34294508

[19] Nguyen AT, Nandi P, Roe AH, Pecker LH, Whiteman MK, Tepper NK, Safety of hormonal contraception among women with sickle cell disease: an updated systematic review. Contraception 2025:111275. 10.1016/j.contraception.2025.111275 41177210

[20] Phillips SJ, Hofler LG, Modest AM, Harvey LFB, Wu LH, Hacker MR. Continuation of copper and levonorgestrel intrauterine devices: a retrospective cohort study. Am J Obstet Gynecol 2017;217(1):57 e1–6. 10.1016/j.ajog.2017.03.005 PMC5810132 28315664

[21] Udeze C, Jerry M, Evans KA, Li N, Jain S, Andemariam B. Clinical and economic burden of managing patients with sickle cell disease receiving frequent red blood cell transfusions in the United States. Clinicoecon Outcomes Res 2025;17:303–13. 10.2147/CEOR.S511996 PMC11998934 40236792

[22] Snyder AB, Lane PA, Zhou M, Paulukonis ST, Hulihan MM. The accuracy of hospital ICD-9-CM codes for determining Sickle Cell Disease genotype. J Rare Dis Res Treat 2017;2(4):39. PMC5709815 29202133

[23] Eisenbrown K, Nimmer M, Brousseau DC. The accuracy of using ICD‐9–CM codes to determine genotype and fever status of patients with sickle cell disease. Pediatri. Blood Cancer 2015;62(5):924–5. 10.1002/pbc.25432 25683555

[24] Early ML, Eke AC, Gemmill A, Lanzkron S, Pecker LH. Severe maternal morbidity and mortality in sickle cell disease in the National Inpatient Sample, 2012–2018. JAMA Netw Open 2023;6(2):e2254552. 10.1001/jamanetworkopen.2022.54552 PMC9896307 36729452

[25] Eissa AA, Tuck SM, Rantell K, Stott D. Trends in family planning and counselling for women with sickle cell disease in the UK over two decades. J Fam Plann Reprod Health Care 2015;41(2):96–101. 10.1136/jfprhc-2013-100763 24860151

