# Supplementary material for: Patterns of hormonal contraception use in women with sickle cell disease and employer-sponsored insurance
Source: Contraception. Author manuscript; Available in PMC 2026 Oct 11. (PMC13654136; doi:10.1016/j.contraception.2026.111399)
Supplement: Supplementary file 1 [file NIHMS2198031-supplement-1.docx]

**Supplemental Table 1: ICD, CPT and HCPCS Codes**

| **Covariate** | **ICD-9 DX Code** | **ICD-10 DX Code** | **CPT Codes** | **ICD 9/10-PCS** | **HCPCS Codes** |
| --- | --- | --- | --- | --- | --- |
| **SCD genotype** | | | | | |
| HbSS | 282.61 282.62 | D57.00 D57.01 D57.02 D57.03 D57.09 |  |  |  |
| HbSC | 282.63 282.64 | D57.20 D57.211 D57.212 D57.213 D57.218 D57.219 |  |  |  |
| HbSβ^+^/β0 Thalassemia | 282.41 282.42 | D57.40 D57.411 D57.412 D57.419 D57.42 D57.431  D57.432 D57.438 D57.439 D57.44 D57.451 D57.452 D57.453 D57.458 D57.459 |  |  |  |
| Non-specific SCD | 282.60 282.68 282.69 | D57.1 D57.80 D57.811 D57.812 D57.813 D57.818 D57.819 |  |  |  |
| **Comorbidities** | | | | | |
| Obesity | 278.00 278.01 V85.30 V85.31 V85.32 V85.33 V85.34 V85.35 V85.36 V85.37 V85.38 V85.39 V85.41 V85.42 V85.43 V85.44 V85.45 V85.54 | E66.01 E66.09 E66.1 E66.2 E66.8 E66.9 Z68.30  Z68.31 Z68.32 Z68.33 Z68.34 Z68.35 Z68.36 Z68.37 Z68.38 Z68.39 Z68.41 Z68.42 Z68.43 Z68.44 Z68.45 Z68.54 |  |  |  |
| Smoking | 305.1 989.84 V15.82 | F17.21 Z72.0 |  |  |  |
| Diabetes | 250.00-250.93 | E08.00-E13.9 |  |  |  |
| Hypertension | 401.0-405.99 | I10-I16.9 |  |  |  |
| Hyperlipidemia | 272.0 272.1 272.2 272.3 272.4 | E78.0 E78.00 E78.01 E78.1 E78.2 E78.3 E78.4 E78.41 E78.49 E78.5 |  |  |  |
| Cancer (non-skin) | 140-171.9 174.0-195.8 200.00-209.36 | C00.0-C41.9 C45.0-C76.8 C7A-00-C7A.8 C81.00-C96.9 |  |  |  |
| **Contraception Related** | | | | | |
| IUD | V25.11 | Z30.014 Z30.430 | 58300 | 697 0UH97HZ 0UH98HZ 0UHC7HZ 0UHC8HZ 0UH90HZ | J7301 J7302 S4989 Q0090 S4981 J7297 J7298 J7296 |
| Copper IUD |  |  |  |  | J7300 |
| Implant | V25.5 | Z30.017 | 11981 | 0JHD0HZ 0JHD3HZ 0JHF0HZ 0JHF3HZ 0JHG0HZ 0JHG3HZ 0JHH0HZ 0JHH3HZ | J7306 J7307 |
| Injectable |  | Z30.013 |  |  | J1050 |
| Patch |  | Z30.016 |  |  | J7304 |
| Ring |  | Z30.015 |  |  | J7303 |
| Removal | V25.12 | Z30.432 | 58301 11976 11982 | 9771 0UPD7HZ 0UPD8HZ |  |

Abbreviations: CPT, Current Procedural Terminology; HCPCS, Healthcare Common Procedure Coding System; ICD, International Classification of Diseases; SCD, sickle cell disease

**Supplemental Table 2: Age and Residence of Privately Insured Reproductive-age Women with SCD, by Study Year (Percentages), MarketScan Database 2012-2019**

| **Year** | **Base N** | **Age (years)** | | | |  | **Region** | | | | **Rural Residence** |
| --- | --- | --- | --- | --- | --- | --- | --- | --- | --- | --- | --- |
|  |  | **12-18** | **19- 26** | **27-34** | **35-44** |  | **Northeast** | **North Central** | **South** | **West** |  |
| 2012 | 2,261 | 27% | 26% | 21% | 26% |  | 21% | 14% | 56% | 7% | 8% |
| 2013 | 2,314 | 26% | 26% | 21% | 27% |  | 23% | 12% | 55% | 8% | 7% |
| 2014 | 2,449 | 25% | 28% | 20% | 27% |  | 24% | 12% | 56% | 6% | 6% |
| 2015 | 1,810 | 23% | 28% | 22% | 27% |  | 20% | 14% | 61% | 5% | 6% |
| 2016 | 1,812 | 23% | 28% | 22% | 28% |  | 19% | 14% | 62% | 5% | 6% |
| 2017 | 1,802 | 22% | 27% | 23% | 28% |  | 19% | 14% | 62% | 4% | 6% |
| 2018 | 1,825 | 22% | 27% | 25% | 27% |  | 22% | 14% | 59% | 5% | 6% |
| 2019 | 1,688 | 22% | 27% | 24% | 27% |  | 23% | 13% | 60% | 5% | 5% |

Age defined as age enrolled in private insurance during respective year. Rural residence is defined as residing in a county that is not classified as a Metropolitan Statistical Area. 5,957 unique women.

Abbreviations: SCD, sickle cell disease
